# Osteopathic Care as (En)active Inference: A Theoretical Framework for Developing an Integrative Hypothesis in Osteopathy

**DOI:** 10.3389/fpsyg.2022.812926

**Published:** 2022-02-18

**Authors:** Jorge E. Esteves, Francesco Cerritelli, Joohan Kim, Karl J. Friston

**Affiliations:** ^1^Clinical-Based Human Research Department, Foundation COME Collaboration, Pescara, Italy; ^2^Malta ICOM Educational, Gżira, Malta; ^3^Research Department, University College of Osteopathy, London, United Kingdom; ^4^Department of Communication, Yonsei University, Seoul, South Korea; ^5^Wellcome Centre for Human Neuroimaging, Institute of Neurology, London, United Kingdom

**Keywords:** osteopathy, active inference, therapeutic alliance, predictive processing, enactivism, free-energy principle, chronic pain, affective touch

## Abstract

Osteopathy is a person-centred healthcare discipline that emphasizes the body’s structure-function interrelationship—and its self-regulatory mechanisms—to inform a whole-person approach to health and wellbeing. This paper aims to provide a theoretical framework for developing an integrative hypothesis in osteopathy, which is based on the enactivist and active inference accounts. We propose that osteopathic care can be reconceptualised under (En)active inference as a unifying framework. Active inference suggests that action-perception cycles operate to minimize uncertainty and optimize an individual’s internal model of the lived world and, crucially, the consequences of their behaviour. We argue that (En)active inference offers an integrative framework for osteopathy, which can evince the mechanisms underlying dyadic and triadic (e.g., in paediatric care) exchanges and osteopathic care outcomes. We propose that this theoretical framework can underpin osteopathic care across the lifespan, from preterm infants to the elderly and those with persistent pain and other physical symptoms. In situations of chronicity, as an ecological niche, the patient-practitioner dyad provides the osteopath and the patient with a set of affordances, i.e., possibilities for action provided by the environment, that through shared intentionally, can promote adaptations and restoration of productive agency. Through a dyadic therapeutic relationship, as they engage with their ecological niche’s affordances—a structured set of affordances shared by agents—osteopath and patient actively construct a shared sense-making narrative and realise a shared generative model of their relation to the niche. In general, touch plays a critical role in developing a robust therapeutic alliance, mental state alignment, and biobehavioural synchrony between patient and practitioner. However, its role is particularly crucial in the fields of neonatology and paediatrics, where it becomes central in regulating allostasis and restoring homeostasis. We argue that from an active inference standpoint, the dyadic shared ecological niche underwrites a robust therapeutic alliance, which is crucial to the effectiveness of osteopathic care. Considerations and implications of this model—to clinical practice and research, both within- and outside osteopathy—are critically discussed.

## Introduction

Osteopathy is a person-centred healthcare discipline that emphasizes the body’s structure-function interrelationship and its self-regulatory mechanisms to inform a whole-person clinical approach to health and wellbeing, traditionally involving primarily manual treatment (European Standards EN 16686, 2015). Although several authors regard it as a unique philosophy of healthcare, which is practised according to distinctive principles (e.g., Cotton, 2013; Paulus, 2013), others have argued that osteopathic principles no longer define osteopathy as a distinctive healthcare profession (e.g., McGrath, 2013; Tyreman, 2013). In fact, Tyreman (2013) argued that osteopathic principles are principles of good patient-centred care in the current world of healthcare. Although osteopathy cannot be a philosophy like, for example, positivism or phenomenology, it can arguably be viewed as a particular conceptualization of what human health and *unhealth* are, one that guides osteopaths’ thinking about patient care (Tyreman, 2018a,b). Within this conceptual framework, the osteopath’s role should go beyond simply explaining and treating, to provide support and insight into the meaning of the patient’s illness experiences to improve their sense of self and consequently their health and wellbeing (Tyreman, 2018b). Despite the claimed person-centeredness of osteopathic care, clinicians have, for long, focused on cause-effect “body-centred,” biomedical care models (Thomson et al., 2013; Tyreman, 2013; Esteves et al., 2020). Osteopaths have traditionally focused on diagnosing and treating somatic dysfunction, and its predecessor’ osteopathic lesion’ (Thomson et al., 2013; Fryer, 2016; Noy et al., 2020). Somatic dysfunction is described as *“impaired or altered function of related component of the somatic (body framework) system: skeletal, arthrodial, and myofascial structures, and related vascular, lymphatic, and neural elements”* (Fryer, 2016). The concept of somatic dysfunction still plays a vital role in osteopathic practice, professional regulation, and professional identity (Fryer, 2016; Alvarez et al., 2020; Nesi, 2020; Santiago et al., 2020; Tramontano et al., 2020, 2022; Cerritelli et al., 2021b). However, an uncritical emphasis on evaluating and treating somatic dysfunctions can be regarded as a biomedical form of clinical evaluation and, arguably, a reductionist activity (Thomson et al., 2013). From an organism’s perspective, the possibility of things going well or poorly is rooted in the organism’s dynamic co-constitution, components, and environment (Ongaro and Ward, 2017). Concentrating on a body part in isolation from these dynamics obscures the properties that make it potentially relevant to the health and viability of the organism (Ongaro and Ward, 2017). Critically, effective person-centred care is highly dependent on developing a solid therapeutic alliance between the patient and clinician, which is influenced by biopsychosocial factors such as age, bodily experiences, expectations, values and beliefs, and personal and socio-cultural attributes (Miciak et al., 2018; Søndenå et al., 2020). A robust therapeutic alliance enables osteopaths to assist patients in making sense of their illness experiences by developing new body narratives about their altered or changing physical capacities (Gale, 2011), as well as the effects on their identity, relationship with their internal and external environment, and sense of meaning in their lives (Tyreman, 2018a). Being truly person-centred requires recognising and critically integrating these factors into osteopathic care for patients (Tyreman, 2018b).

Recent advances in neuroscience, cognitive science and philosophy, pain science, and musculoskeletal care, all of which support osteopaths’ proponents of person-centred care, present an unprecedented opportunity to develop and disseminate evidence-based models of osteopathic care (Esteves et al., 2020). We would argue that osteopathic concepts of self-regulation, adaptation, sense of agency, and an individual’s capacity to engage with—and act on—their environment (Tyreman, 2018a; Vogel, 2021) are consistent with an ecologically enactive approach to mind and life. These ideas are not entirely new in osteopathy and are, arguably, aligned with Hoover’s (1963) perspective on osteopathy as ecological medicine. In fact, he argued that Andrew Taylor Still’s original ideas were centred around addressing changes that interfered with an individual’s function and their impact on their daily life (Hoover, 1963). However, to achieve worldwide statutory regulation and recognition, osteopathy moved closer to allopathic medicine and the biomedical care model (Hoover, 1963). An ecologically enactive approach to mind and life is founded on the Free Energy Principle and its corollary, active inference theory (Friston, 2009; Bruineberg et al., 2018). Indeed, some have argued that we should view agency and the sense of agency through the lens of the Free Energy Principle (Bruineberg, 2017). According to enactivism, cognition and perception develop due to a dynamic interaction between an acting organism and its environmental constraints, referred to as affordances (Thompson, 2010; Tschacher et al., 2017)—affordances are opportunities for action, e.g., a door for opening or a ball for catching, rather than an action-independent representation of the “way things are” (Seth, 2021). The mind, body, and environment are, therefore, highly interdependent elements of an ecological system (Tschacher et al., 2017). A fundamental notion of enactivism is sense-making—the evaluative interaction of an organism with its environment (De Haan, 2020b). Recently, Stilwell and Harman (2019) have proposed that pain should be regarded as a relational and emergent process of sense-making through a lived body, which cannot be separated from the world that we shape and that shapes us. Interestingly, Littlejohn (1905), in his early conceptual framework for osteopathy, focused on the functional adaptation of the body in relation to the external environment. He viewed osteopathy as person-centred care, which is based on four key pillars: adaptation, function, environment and immunity (Gevitz, 1982). Although many of these early osteopathic care concepts were lost to a predominantly cause-effect disease-based model, we argue that these ideas can be reconciled under the Free Energy Principle (FEP) and the enactivist and active inference frameworks.

Humans are an example of a complex and dynamic adaptive system, and this should inform the osteopath’s clinical reasoning process. Where is the breakdown in the patient’s adaptive capacity, and how can osteopathy improve their adaptability are some of the questions that osteopaths face in their reasoning and decision making. The FEP explains how dynamic adaptive systems maintain their integrity, i.e., non-equilibrium steady-state, by restricting themselves to a limited number of characteristic states (Hipolito, 2019). Any adaptive change made by an organism or biological system must therefore minimize its long-term average surprise, where surprise scores the implausibility of a system being in a particular state (e.g., it would be surprising to find a fish out of water). Mathematically, this amounts to minimizing the dispersion or entropy of sensed states, because surprise (a.k.a. surprisal) is self-information. Clinically, it mandates the mitigation of unpredicted and uncharacteristic sensations (Edwards et al., 2012). The long-term average of surprise is associated with the entropy (dispersion) of sensations: a failure to minimize surprise would therefore lead to an unbounded increase in entropy (sensory disorder) and dissolution of self-organization and consequent homeostasis (Edwards et al., 2012). Living systems typically resist a natural tendency to disorder by minimizing surprise and uncertainty by acting on the world and updating their internal states—through active inference (Friston, 2009; Ramstead et al., 2019)^1^. This active inference can be read as selecting the most likely course of action, under an internal narrative or generative model of the world (and body) that covers the consequences of action. A breakdown in adaptive capacity of the person seeking care due to an inflexible or distorted updating of such models will lead to illness. A robust therapeutic alliance may be necessary for healthy adaptation—by facilitating a revision of their generative model or narrative that renders it apt for changes their world (and body).

Active inference foregrounds the crucial predisposition of living organisms to adapt by creating, updating, and maintaining inferences about their environment (Bouizegarene et al., 2020). This is fundamental in the context of osteopathy, and despite the still prevailing views that structural abnormality causes functional disorder (Tyreman, 2013), it is clearly aligned with osteopathic concepts of adaptation (Littlejohn, 1905; Vogel, 2021). Active inference casts sentient behaviour as the selection of action policies (i.e., sequences of active states) that minimize expected free-energy, in order to reduce expected surprise. Acting to minimise expected surprise can be read as acting to resolve uncertainty, which equips the affordance—of any action on the world with an—epistemic aspect (Ramstead et al., 2019). In short, according to active inference, biological agents act to fulfil prior beliefs (installed in a generative model) about the consequences of action to ensure that the agent actively avoids surprising states (Friston, 2009). Active inference generalizes Bayesian inference beyond inferring the latent or hidden states that cause sensations, to act in a way that minimizes expected surprise (i.e., uncertainty). To this end, action selection and choice behaviour can be viewed as inferring the policies that minimize the free-energy expected on pursuing that policy (Friston et al., 2020). This is sometimes known as planning as inference. Active inference can therefore be regarded as the process that confirms and updates the evidence for the generative model that a living system entails and enacts in living (Ramstead et al., 2019); also known as self-evidencing (Hohwy, 2016).

This paper aims to provide a theoretical framework for developing an integrative hypothesis in osteopathy, which is based on the enactivist and active inference accounts. In osteopathy and other professions, evidence has undermined existing biomedical or biomechanically based models of care, resulting in the need to reconceptualise patient evaluation and treatment strategies (Gliedt et al., 2017; Jull, 2017; Thomson and Abbey, 2017; Smith, 2019). Therefore, it is crucial that osteopaths and other healthcare professionals critically consider relevant research that can assist in developing an integrative model of care that focuses on the whole person rather than on the mechanisms operating within the body (Tyreman, 2018b). Enactivism and active inference have recently been proposed as robust theoretical frameworks to understand, for example, pain (Tabor et al., 2017, 2020; Pezzulo et al., 2019; Stilwell and Harman, 2019; Tabor and Burr, 2019; Coninx and Stilwell, 2021; Kiverstein et al., 2021), patient care in psychiatry (De Haan, 2020b), the development of a therapeutic alliance (e.g., Connolly, 2022), and placebo (Ongaro and Ward, 2017; Ongaro and Kaptchuk, 2019). We propose that osteopathic care can be reconceptualised under (En)active inference as a unifying framework. Active inference suggests that action-perception cycles operate to minimize uncertainty and optimize an individual’s internal model of the lived world and, crucially, their behaviour. We argue that (En)active inference offers an integrative framework for osteopathy, which can evince the mechanisms underlying dyadic and triadic (e.g., in paediatric care) exchanges and osteopathic care outcomes. We propose that this theoretical framework can underpin osteopathic care across the lifespan, from preterm infants to the elderly and those with persistent pain and other physical symptoms. In situations of chronicity, as an ecological niche, the patient-practitioner dyad provides the osteopath and the patient with a set of affordances that, through shared intentionally, can promote adaptations and restoration of productive agency (Ramstead et al., 2019). Underpinned by a robust therapeutic alliance, osteopaths help patients make sense of their illness experiences by creating new body narratives about their changed or changing physical capacities and ensuing effects on their identity, relationship with their environment and meaning in their lives. Therefore, through a dyadic therapeutic relationship—a structured set of affordances—osteopath and patient actively construct a shared, sense-making narrative and realize a shared generative model of their engagement with their ecological (therapeutic) niche (Ramstead et al., 2019). In general, touch plays a critical role in developing a robust therapeutic alliance, mental state alignment, and biobehavioural synchrony between patient and practitioner. However, its role is particularly crucial in the fields of neonatology and paediatrics, where it becomes central in regulating allostasis and restoring homeostasis (Mc Parlin et al., 2022). We argue that from an (En)active inference standpoint, the shared ecological niche underwrites a robust therapeutic alliance, which is crucial to the effectiveness of osteopathic care and other forms of manual therapy.

## Osteopathic Care Across the Lifespan

Since its inception in the late nineteenth century in the United States of America, osteopathy has developed as two distinct healthcare disciplines—osteopathic physicians and osteopaths. Despite significant differences in their scope of practice, both osteopaths and osteopathic physicians provide patient care informed by concepts of body unity, structure-function relationships, and self-regulation to facilitate adaptation and maintain homeostasis. Despite the osteopathic principles’ purported distinctiveness (e.g., Cotton, 2013; Paulus, 2013), these concepts are widely accepted in various fields of science and healthcare—for example, structure-function interrelationship is a widely accepted biological principle (e.g., Bojar, 2020). Moreover, they are fully aligned with contemporaneous principles of patient-centred care (Tyreman, 2018b; Esteves et al., 2020; Vogel, 2021). However, an essential distinction of osteopathic care is that it is health-centred rather than disease-centred, emphasising the therapeutic relationship’s person-centred nature (Kuchera, 2018). This emphasis on health over disease is reflected in Andrew Taylor Still’s writings on the science and practice of osteopathy —*“To find health is the object of the doctor. Anyone can find disease”* (Still, 1902, p. 72).

Osteopaths care for patients of all ages who present with a variety of clinical conditions. Although the majority of evidence for osteopathic manipulative treatment focuses on musculoskeletal disorders such as low back pain (Franke et al., 2014) and neck pain (Franke et al., 2015), there is also evidence of its effectiveness on migraine patients (Cerritelli et al., 2017) and preterm infants (Lanaro et al., 2017). Despite these encouraging findings, the effectiveness of osteopathic manipulative treatment in managing musculoskeletal conditions is highly variable. For instance, the results of a recent well-conducted clinical trial in patients with non-specific low back pain demonstrated that standard osteopathic treatment had a negligible effect on low back pain-specific activity limitations compared to sham osteopathic treatment (Nguyen C. et al., 2021). While one could argue that the recent clinical trial results are not clinically meaningful, they nonetheless demonstrate that a sham touch-based intervention improved pain outcomes and absenteeism at work. Although some would argue that these results may be explained by expectation-associated placebo effects, in individuals with chronic pain, the alleviation of symptoms with placebo treatment is more likely attributed to non-conscious Bayesian biases (Rossettini and Testa, 2018; Kaptchuk et al., 2020). Beyond traditional mind-body dualism, predictions are already encoded at the very instant of transduced exteroceptive, interoceptive and proprioceptive sensory data associated with bodily symptoms and the standard or sham therapeutic intervention (Kaptchuk, 2018; Ongaro and Kaptchuk, 2019). Crucially, *“the body understands and is capable of responding to meaning without conceptual or linguistic content specified”* (Frenkel, 2008). In other words, the organism’s embodied regulatory systems need to make an inference about the viable states of that organism to minimize surprisal and maintain homeostasis (Bruineberg, 2017). We would, therefore, argue that a pleasant manual intervention delivered within the context of a positive therapeutic encounter, which is underpinned by effective verbal and non-verbal communication strategies, is apt to facilitate the revision of generative models in the following sense. The therapeutic setting provides sensory evidence in all the sensory modalities necessary to update a generative model of the embodied self. Crucially, these not only include the usual exteroceptive cues that allow the patient to infer intentional stance of the therapist but, crucially proprioceptive and interoceptive (e.g., affiliative touch) evidence that pertains directly to the patient’s body and its responses. Although touch is central to osteopathy and other healthcare professions such as physiotherapy, chiropractic and nursing, evidence from the field of musculoskeletal care demonstrates that other environmental elements or exteroceptive cues (aka “contextual factors”) as verbal and non-verbal communication within the patient-practitioner dyad, can nonetheless shape the context aimed at updating the patient’s generative model (Palese et al., 2019; Rossettini et al., 2020; Thomson and Rossettini, 2021).

Despite the challenges associated with the nature and strength of clinical evidence regarding the effectiveness of osteopathic manipulative treatment for musculoskeletal disorders, little is known regarding the impact that touch is having on the patient’s nervous system during osteopathic procedures and its impact on, for example, pain modulation, autonomic nervous system function and emotional processing (McGlone et al., 2017). In a study investigating the effect of 5 min of CT afferent optimal velocity stroking touch to 5 min of static touch on the heart rate and oxygen saturation levels on a cohort of preterm infants between 28- and 37-weeks gestational age, Manzotti et al. (2019) found that CT afferent touch produced a significant decrease in infants’ heart-rates and increase in their blood oxygenation levels, thus reducing their autonomic arousal. In a cohort of asymptomatic adults, Tamburella et al. (2019) found a decrease of resting cerebral perfusion immediately after osteopathic treatment in a cluster of brain regions comprising the Posterior Cingulate Cortex and the Superior Parietal Lobe; while Posterior Cingulate Cortex perfusion increased significantly after 3 days post-osteopathic treatment. Given the Posterior Cingulate Cortex key role in the Central Autonomic Network, the authors speculate that the observed change in its perfusion might indicate an osteopathic dependent sympathovagal modulation (Tamburella et al., 2019). In another neuroimaging study in patients with chronic low back pain, the osteopathic treatment led to a distinct and specific reduction in cerebral perfusion in specific interoceptive areas—bilateral insula, anterior cingulate cortex, left striatum and right medial frontal gyrus (Cerritelli et al., 2020). The changes observed in the insular cortex, corroborate the hypothesis previously proposed that osteopathic treatment might exploit an interoceptive paradigm, which may explain some of its clinical effects (D’Alessandro et al., 2016). Finally, recently published data from the same cohort of chronic low back pain sufferers, revealed that the left posterior insula is a specific target of the osteopathic treatment effect (Cerritelli et al., 2021a). The authors argue that osteopathic care might produce a significant change in cerebral activity, specifically on key areas related to the Central Autonomic Network and therefore to interoception and interoceptive control (Cerritelli et al., 2021a). Taken together, the results of these studies across the lifespan demonstrate osteopathic and touch specific neurobiological correlates that could arguably be interpreted through the lens of (En)active inference, i.e., considering the whole person and the dynamic interactions with their environment. Crucially, the interactions with the environment which enact the patients’ experiences are dependent on contextual factors and communication between patient and practitioner. From a predictive processing standpoint, cues in the environment and personality variables are likely to shape the perception of touch even before the touch occurs (Sailer and Leknes, 2022). Geri et al. (2019) have argued that in physiotherapy, the practitioner’s hands, while meeting the patient’s expectations, also provide the vehicle to non-verbally communicate meaningful signals to the patient’s brain, modulate their pain and regulate their emotions. Although touch plays a central role in osteopathy and manual therapy in general, therapeutic interventions should be framed within a contemporary multidimensional, neurophysiologically based (and arguably psychologically informed) model of care, which avoids dependence on a predominantly passive approach (Rabey et al., 2017). We argue that clinicians could critically consider the value of an (En)active inference framework that does not focus on one system or component of a model (e.g., biological, psychological or social factors) in isolation. Person-centred care should be truly focused on the whole organic person rather than on the mechanisms operating within the body (Tyreman, 2018b). In osteopathy, there are positive attempts to integrate existing models of care in a person-centred framework (e.g., Smith, 2019; Castagna et al., 2021). Notwithstanding these positive developments, empirical research is needed to develop further and validate these putative conceptual frameworks.

### Bayesian Brain, Persistent Physical Symptoms and the Sense of Self

#### Predictive Coding, Predictive Processing, and the Bayesian Brain

Predictive coding, which has its origins in the work of Helmholtz (1867), is based on the premise that a system, such as the brain, possesses an internal model of the causes of its sensory input. What we perceive is always a delicate balance of top-down knowledge-based prediction and bottom-up sensory input (Clark, 2013). Thus, assuming that its current perceptual beliefs are accurate, the perceptual system repeatedly predicts what it should observe next (Friston, 2005; Kiebel et al., 2008; Bastos et al., 2012; Smith et al., 2021). Predictive coding updates perceptual beliefs when there is a discrepancy between sensory evidence and predicted sensory input; this discrepancy allows for the calculation of a “prediction error” (Smith et al., 2021). In predictive coding theory, prediction errors are used to guide belief updating and determine the smallest change in perceptual beliefs necessary to minimize error signals; this is referred to as Bayesian belief updating or inference (Smith et al., 2021). Notably, predictive coding is primarily concerned with making predictions about the connections between sensory evidence and the generative model based on the current state of the world rather than with anticipating the future. Much of the work on predictive coding has been absorbed into the more general theory of predictive processing. Predictive processing has emerged as a leading theoretical framework in cognitive science and philosophy of mind over the last few years (Hohwy, 2020). In effect, it basically extends the predictive coding paradigm to cover the consequences of action—moving from purely perceptual inference to active inference.

According to predictive processing, the brain is a homeostatic machine that is primarily capable of making sub-personal (Bayesian) statistical inferences (Clark, 2013). On this view, the brain is a hierarchical, multilevel predictive machine whose primary function is to anticipate the distal causes of internal and external sensory stimuli and minimize the discrepancy between the prediction and the received stimulus’s occurring signal (Clark, 2013, 2015). Crucially, these causes include action; either motor action or autonomic action, causing changes in proprioceptive and interoceptive sensations, respectively. Thus, perception is an active process: brains predict and test their hypotheses against incoming sensory evidence rather than merely reacting to the world (Friston, 2009). Anticipation and action consist of making bidirectional inferences. At hierarchically lower-levels of processing, the brain uses its generative model to make predictions about the incoming interoceptive, proprioceptive and exteroceptive input (Friston, 2005; Smith et al., 2019). When the sensory input does not match the prediction, the lower-level levels broadcast a prediction-error signal to the upper levels of neural processing that drive changes in the generative model (Clark, 2013; Smith et al., 2019). In the upper levels of neural processing (e.g., secondary sensory and association cortices), by updating its generative model, the brain better predicts how the lower-level inputs will change; thereby resolving lower-level prediction errors (Friston, 2009; Clark, 2013; Smith et al., 2019). Differences between the predictions and the lower-level expectations are calculated, and only residual “prediction errors” are sent forward (and sideways) to generate better forecasts to drive inference and learning (Clark, 2013, 2015). In short, the minimization of prediction error can be accomplished through *perceptual inference* and *active inference*. Whereas perceptual inference involves updating the generative model based on prediction errors; active inference is a process whereby the agent acts on the world to create the sensations it predicts or anticipates (Venter, 2021). In sum, active inference offers an expressive framework within which to understand *health* and *unhealth* across the lifespan and, arguably, in providing person-centred osteopathic care.

#### The Self and the Sense of Agency

**FIGURE 1:**
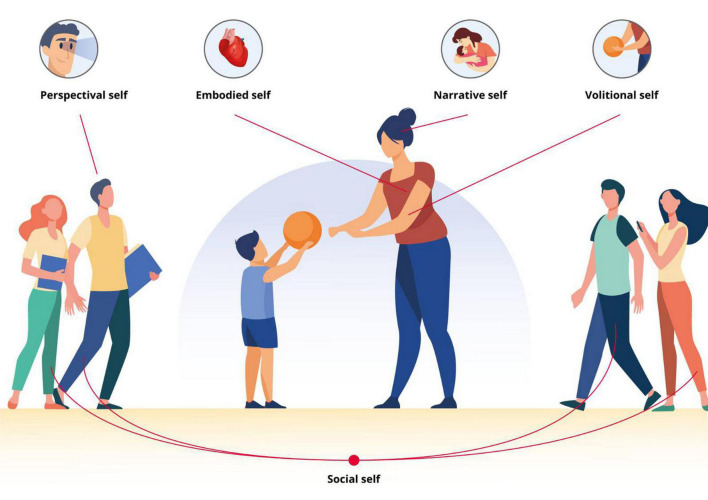
Multilevel representation of human selfhood according to Seth (2021). *Embodied self*—the sense of having a body, *perspectival self*—the ability to perceive the world from a first-person perspective, *volitional self*—the sense of agency, *narrative self*—the sense of personal identity, *social self*—linked to the way in which we are perceived by, and interact with others.

We do not need to think about our embodied organism when we are healthy—our “invisible” bodies provide us with familiar, sensorial pleasures through our daily activities and interactions with the environment and others (Arika, 2019). Our brain uses the body to sample and make sense of sensory input, thus contributing to our sense of self. Therefore, brains are embodied—they communicate with their environment *via* an “invisible” body. Moreover, the brain is embedded in and in constant interaction with its environment—an environment that often contains other embodied brains (Seth, 2021). One could therefore argue that a brain that is not embodied and embedded in the natural world would be incapable of supporting the phenomenologically rich and detailed experiences that we typically enjoy (Kirchhoff and Kiverstein, 2019). Although *“our conscious experiences of the world and the self are forms of brain-based predictions, which arise with, through, and because of our living bodies”* (Seth, 2021), the sense of self is a deeply phenomenological, multilevel perception. Crucially, human selfhood includes the *embodied self*—the sense of having a body, *the perspectival self*—the ability to perceive the world from a first-person perspective, *the volitional self*—the sense of agency, *the narrative self*—the sense of personal identity, and *the social self*—linked to the way in which we are perceived by, and interact with others (Seth, 2021; Figure 1).

While this multilevel sense of self is necessary for understanding who we are and how we feel, human life is largely defined by goal-oriented activities within an environment. Crucially, we are agents—or actors—who, through our participation, shape our reality. Rather than perceiving ourselves to know ourselves, we perceive ourselves to control ourselves (Seth, 2021). As agents, our conscious experiences are thus formed through direct interaction with—and exploration of—the environment (Kirchhoff and Kiverstein, 2019). We anticipate ourselves into existence. Thus, agency can be defined as an organism’s or system’s capacity to perform goal-directed or intentional actions—agency is demonstrated whenever an individual acts independently in their environment or when they act under specific goals (Kauffman, 2000; Barandiaran et al., 2009; Segundo-Ortin, 2020). Therefore, an agent should model itself as an active agent capable of selectively interacting with its environment (Bruineberg, 2017). This implies a sense of agency, i.e., the ability of the system to infer the sensory consequences of an action based on prior experiences (Friston et al., 2013; Deane et al., 2020). Although action entails specific types of movement, it is more than that (Gallagher, 2020). Not all actions are conscious, but they can be embodied and unconscious. Importantly, in a state of health and “invisibility” of the body, the sense of agency and self do not require the agent to consciously attend to the motor details of their own bodily movements as they are engaged in action (Gallagher, 2020). From an enactivist perspective, perception and cognition are action-oriented—therefore, when one perceives something, perceives it as actionable, i.e., something one can reach or not; or something one can pick up or not (Gallagher, 2020). The ability to operate as agents, thus creating our reality through anticipation and participation, is a crucial factor that undergirds our physical and emotional wellbeing. Through this lens, osteopaths make a professional judgment about where the person’s relationship with their environment has broken down and what is preventing them from realising full productive agency (Tyreman, 2018a). The sign of becoming well is to regain agency and take one’s part in the world again.

#### Persistent Physical Symptoms Through the Lens of Predictive Processing

Persistent pain is a pervasive, complex, and distressing problem for individuals and society, accounting for the majority of disability and disease burden globally (GBD 2016 Disease and Injury Incidence and Prevalence Collaborators, 2017; Mills et al., 2019). Despite persistent pain’s global prevalence, its causes and underlying mechanisms remain unclear. A growing body of evidence indicates that persistent pain is a complex web of sensory and emotional experiences coupled with behavioural adaptations (Vachon-Presseau et al., 2016). In the case of chronic low back pain, evidence indicates that the transition from acute to persistent pain is facilitated by disruptions in the brain reward system’s mesocorticolimbic circuitry (Vachon-Presseau et al., 2016; Yu et al., 2020). In persistent pain states, the brain has learned to filter emotions, actions, and reward through the lens of pain, causing the brain to develop an addiction to pain (Vachon-Presseau et al., 2016). As a result, effective treatment strategies must address this maladaptive neuroplasticity. Although a recent neuroimaging study demonstrated beneficial effects of osteopathic treatment on the interoceptive system of patients with persistent low back pain (Cerritelli et al., 2020, 2021a), additional research is necessary to fully understand the role of osteopathic care in reversing chronic pain-related maladaptive neuroplasticity. Indeed, it is improbable that osteopathic care models centred on the “body” would successfully address the conundrum of persistent pain and other physical symptoms. Many believe that biopsychosocially informed person-centred care is a viable solution to the problem. On the other hand, the biopsychosocial model remains ambiguous in its ability to precisely describe the nature of interactions between the various systems (De Haan, 2020a,b). As a result, osteopathic care must be conceptualised in terms of the agent’s relationship to their internal and external environments.

While the body “disappears” in states of health and wellbeing, it typically “reappears” at times of pain and dysfunction (Leder, 1990, p. 4). Therefore, physical or emotional pain affects the very foundation on which the sense of self rests (Arika, 2019). The physiological arousal, which occurs in persistent pain and other persistent physical symptoms, prompts the individual to focus attention on their body (Van den Bergh et al., 2017). In this context, pain and other physical symptoms should be viewed as an action problem—when a nociceptive signal travels up from the periphery *via* the spinal cord, it presents the brain with the question, “what is to be done”? (Morrison et al., 2013). The nervous system is organized to anticipate potential pain and adjust behaviour before tissue damage becomes critical. Regulatory processes occur dynamically at different levels and in a Bayesian way, i.e., using previous experiences as they are represented in the brain as an estimate of the likelihood that a specific clinical condition applies (Morrison et al., 2013; Van den Bergh et al., 2018). A critical point in cases of pain and dysfunction is that the body does not simply become “visible”—it becomes the focus of attention. This selective attention to the body disrupts the individual’s ability to interact with the environment and others, i.e., their sense of agency. Arguably, illness becomes a loss of agency—the person’s inability to perform goal-oriented actions in the usual expected way marks the beginning of becoming a patient. In predictive processing formulations of active inference, the deployment of attention is generally thought of as covert action. Technically, attention boosts certain prediction errors thereby increasing their influence on belief updating at higher hierarchical levels. This boost rests on an estimation of the precision or confidence that can be placed certain prediction errors. In other words, prediction errors are weighted by a prediction of predictability. Many active inference formulations of chronic pain emphasise this attentional aspect. Put simply, chronic pain represents the hypothesis *“I am in pain”—*a hypothesis that is verified by selectively attending to appropriate sources of sensory evidence; primarily, in the interoceptive and nociceptive domain. Expressed in this way, therapeutic revision of a self-model rests on exploring alternative hypotheses (i.e., self-models) that generate a different attentional set—and a different precision weighting of prediction errors.

On this view, pain and “illness” are not attributes of sensations, but they are carefully crafted narratives over long periods of suffering and engagement with one’s body and healthcare practitioner. They are the best explanations at hand for what one is experiencing. When one thinks of pain or dysfunction, it is not so much the content and prior beliefs that underwrite their commitment to their narrative that they suffer from chronic pain. Instead, it is the fact that they cannot attend away from the information or the sensory evidence that has to be explained in that way (Edwards et al., 2012). Individuals with persistent pain and other physical symptoms are unable to ignore, attend away or attenuate selectively different sources of sensory evidence in order to deploy precision in the context of selective attention or to attenuate or augment it in the context of sensory attenuation (Friston, 2009; Edwards et al., 2012; Pareés et al., 2014). Crucially, individuals have to decide what to attenuate or what to attend to actively. From a precision weighting standpoint, attention refers to the optimization of the precision of prior beliefs regarding the causes of sensory data concerning the precision of those data (Adams et al., 2013; Vasil et al., 2020). Attentional selection is complemented by the attenuation of precision, known as sensory attenuation, i.e., attending away from or ignoring certain sensations, particularly the ones produced by ourselves (Vasil et al., 2020). The attenuation of sensory precision corresponds effectively to attending away from the consequences of action (Edwards et al., 2012; Brown et al., 2013; Pareés et al., 2014). From a predictive coding perspective, sensory attenuation is considered a particular case of optimizing sensory data precision thought to be encoded by the gain of neuronal populations encoding prediction errors (Friston and Frith, 2015). Sensory attenuation can be illustrated by our inability to perceive optical flow produced by saccadic eye movements. When visual motion or flow is produced exogenously, for example, by gently palpating the eyeball, they can be perceived; however, the same does not occur when produced by oculomotor action. This is known as saccadic suppression.

Our brain’s predictions rest on internal models of the body in the world, which are constructed *via* Bayesian inferences constrained by sensory inputs, from which all perceptions and actions emerge (Barrett et al., 2016). When faced with persistent pain and other physical symptoms, the brain has to produce an account of the information that is generated by somatic input (Van den Bergh et al., 2018). Symptoms arise from unconscious inferential processes concerning the nature of interoceptive sensations, while taking into account implicit beliefs and actions—this induces a sense of being real, regardless of the actual somatic input (Henningsen et al., 2018a,b). Consequently, the relationship between symptoms and bodily dysfunction can be highly variable. This relationship may be completely absent in conditions that are typically characterised by highly precise prior expectations and low precision unpredicted somatic input (Henningsen et al., 2018a,b). Arguably, persistent pain and other physical symptoms are the results of a mismatch between the prediction of pain (= p(pain | sensation)) and the likelihood of pain (= p(sensation | pain)). Individuals tend to interpret harmless—or irrelevant—sensations as the consequences of their symptoms and suffering. It is not a failure to shift attention away from the sensation that is a meaningless noise (Hechler et al., 2016). It is instead a failure of dis-attention than a failure of attention—a loss of ability to attenuate. It is a loss of capacity to render one’s body as invisible. Thus, bodily symptoms should be considered in the context of *“action and attention selection dynamics”* (Pezzulo et al., 2019).

In patients with persistent pain and other physical symptoms, a growing number of authors propose that predictive processing models are ideally suited to explain the unique individual experience and hence provide truly patient-centred care (Van den Bergh et al., 2017; Ongaro and Kaptchuk, 2019). It has been argued that the application of predictive processing theory to symptom generation and bodily distress promises to bridge the gap between epistemology and mechanistic common-sense approaches—they eliminate the idea of a homunculus in the mind, which does the perceiving, and replacing it with statistical processes of predictions based on likelihood estimation (Van den Bergh et al., 2018). However, pain is embodied and embedded and represents a protective mechanism—i.e., an action problem—which emerges from ongoing interaction between the body and the world (Tabor et al., 2017, 2020). Therefore, in the context of persistent physical symptoms, it is crucial that models of care based on predictive coding mechanisms also consider the role of active inference in symptom perception and the underlying psychopathological conditions (Pezzulo et al., 2019).

### Touch and Osteopathy in Early Life and Childhood - Shaping the Sense of Self and Agency

The first years of life are marked by rapid brain development and learning. It is challenging for young infants to develop the abilities necessary to navigate their complex environment. They must make sense of their actions by observing the proprioceptive effects of their body movements. Simultaneously, they must develop a fundamental understanding of the behaviour of physical and social entities in their environment. On a physiological level, this learning process is based on the continuous formation and pruning of connections in neuronal networks, which enables infants to interpret sensory information and translate it into appropriate behavioural responses increasingly sophisticatedly. Improved causal learning (Lagnado and Sloman, 2002), self-other distinction (Jeannerod, 2004; Tsakiris et al., 2007), and social and moral interactions are all possible as a result of this capability (Caspar et al., 2016). Infants can learn to use their movements to perform coordinated, intentional actions within a given environment by developing a sense of agency and using their bodies to accomplish goals at an even more fundamental level.

The literature abounds with evidence that human infants construct probabilistic models to represent statistical regularities in their environment (Ruffman et al., 2012; Gopnik and Bonawitz, 2015). Early accounts of motor control appeal to predictive processing and prediction errors in the form of comparator models (Blakemore et al., 2002). Sensory prediction is believed to be based on efferent signals (i.e., the motor command) and is compared to afferent sensory signals. According to the (updated) comparator model theory of agency, whenever the predicted and actual outcomes “match” (i.e., are congruent), this serves as a cue for babies to experience a sense of agency (Zaadnoordijk et al., 2019). The degree of congruence vs. incongruence between predicted and actual sensory outcomes is critical for “experienced” agency (Sato and Yasuda, 2005). It appears, therefore, reasonable to assume that the infant’s brain uses statistical learning principles to make predictions about basic environmental contingencies, which is necessary for the predictive processing framework’s fundamental assumptions about the formation and optimization of predictive models.

The neonatal period is a time of significant neurodevelopment, and thus a time when the level and type of social interaction may significantly influence future social behaviour (Porges and Furman, 2011; Meredith, 2015) and internal models. Touch is critical for nurturing and attachment during development (Walker and McGlone, 2013, for a review) and many social and dyadic interactions in adulthood, with well-documented benefits to health and well-being (House et al., 1988; Berscheid, 2003). Studies demonstrate that tactile nurturing interactions in the neonatal period on adult behaviour and sensitization to neuropeptides (such as oxytocin and arginine vasopressin) positively influence affiliation, aggression, sociosexual behaviour, parental behaviour, and stress reactions (Cushing and Kramer, 2005). As new-born infants and caregivers are in close physical proximity, this increases their growth and development through numerous physiological, behavioural, and neuropsychological metrics (Spitz, 1945; Harlow and Harlow, 1962; Kuhn and Schanberg, 1998).

There has been increasing interest in and growing use of various touch-based therapies over the past half-century. This growing interest and clinical use are supported by substantial research showing the benefits of touch and massage during pregnancy and in the postpartum period (Harrison et al., 2000; Guzzetta et al., 2009; Procianoy et al., 2010; Parashar et al., 2016; Manzotti et al., 2019). Despite advances in understanding how touch is connected to perinatal care, solid evidence is still lacking, and findings are conflicting.

Arguably, osteopathic diagnosis and care and the associated therapeutic relationships between clinicians and their patients depend sensitively on a patient’s and therapist’s sense of touch. Despite the centrality of touch in osteopathy, little is known about the effects of touch on the infant’s nervous system during osteopathic procedures in both early life and childhood. Recently published research provides preliminary evidence on the effect of osteopathic treatment on preterm infants’ heart rate and oxygen saturation (Manzotti et al., 2020), thus highlighting the putative role of osteopathic care in modulating physiological parameters such as autonomic function in the new-born. Considering that the primary function of the nervous system is to manage allostasis, i.e., predicting the physiological needs for survival (Barrett, 2020), these recent research findings may suggest a putative role for osteopathic care in supporting allostatic regulation in preterm infants.

Considering that touch, in all its manifestations, may be regarded as a direct interaction channel with the infant, who may use touch to explore their external and internal environment in novel ways, we can hypothesize that by modifying proprioceptive and interoceptive sensations, generative (self) models are modified and updated, thereby affecting the development of the sense of self. The models are then compared to environmental predictions, updating the sense of agency, and ultimately generating actions that resolve uncertainty about the updated model.

### Beyond Body-Centred Models of Care: Enactivism and Sense-Making

Human beings possess an ever-changing capacity to adapt to their environment. Crucially, each one of us creates our own *Umwelt* (an environment or “life-world” that is unique to us) as a combined creature-environment “bubble” out of those features perceived to be uniquely relevant to its own purposes (Tyreman, 2018a). Therefore, the dynamical interplay of causal factors, the person and their own *Umwelt* make the prediction of illness and dysfunction difficult—attributing cause and effect can be challenging. As such, delivering high quality person-centred osteopathic care is arduous and requires a deeper understanding of the patient as a dynamic, complex adaptive system. Osteopathy cannot simply be conceptualised as a body-centred intervention informed by aetiological models of care: human functioning is complex, unique to the person and unpredictable. Rather than considering their individual patient’s clinical presentation as a set of complex aetiological cause-effect relationships, health and disease should be seen in relation to life and the person within their environment (Hoover, 1963; Tyreman, 2018a). Osteopaths should therefore evaluate the person seeking care within an inconstant ecological system (Tyreman, 2018a).

Despite the complexity and unpredictability of human selfhood and function—which led Hoover (1963) to propose that osteopathy should be regarded as ecological medicine—osteopaths have long focused on the fallacy that removing a structural cause of dysfunction could cure disease. This aetiological model is, for many, an attractive way of approximating osteopathy from orthodox medicine. However, it has been argued that it is far from what Andrew Taylor Still originally envisaged for osteopathy—a way of addressing changes that interfered with an individual’s function and their impact on their activities of daily living (Hoover, 1963). In recent years, several attempts have been made to move away from heavy reliance on aetiological structure-function models of care, by endorsing the biopsychosocial model as the foundation for person-centred osteopathic care (Penney, 2013; Thomson et al., 2013; Esteves et al., 2020). Notwithstanding the centrality of the biopsychosocial model in contemporary healthcare practice, the model does have its own limitations. It has been argued that the biopsychosocial model has been bio-medicalised, lacks a framework that integrates all dimensions in a non-reductionist manner, and it fails to show how its dimensions interrelate (Stilwell and Harman, 2019; De Haan, 2020a,b). To address these limitations of the biopsychosocial model, an enactive approach to acute and chronic pain and mental health disorders has been proposed (Stilwell and Harman, 2019; De Haan, 2020a,b; Coninx and Stilwell, 2021). In line with these recent developments, we have also recently proposed enactivism as a robust framework to underpin the development of an integrative model for person-centred care in osteopathy (Bohlen et al., 2021; Zegarra-Parodi et al., 2021).

Enactivism is a perspective in embodied cognitive science, commonly known as being part of the 4E approaches which argue that mental processes are *embodied*, *embedded*, *enacted*, and *extended* (Rowlands, 2010; Thompson, 2010; Arandia and Di Paolo, 2021). Enactivism rejects an inner-outer division of mind and world and therefore considers organisms as co-arising and co-determining with their environment. As such, organisms and their world are dynamically coupled, i.e., living beings rely on a constant exchange with their environment to maintain themselves (De Haan, 2020a,b). The enactive paradigm is defined by five core concepts—*autonomy*, *sense-making*, *embodiment*, *emergence*, and *experience* (De Jaegher and Di Paolo, 2007). For our thesis, we will briefly focus on two critical concepts: *Autonomy* and *sense-making.*

*Autonomy* is a necessary and sufficient condition to speak of an individual system (Varela, 1979; Di Paolo, 2005; Di Paolo and Thompson, 2014; Segundo-Ortin, 2020; Arandia and Di Paolo, 2021). In systemic terms, autonomous systems such as organisms are defined as operationally closed and precarious networks of mutually enabling processes. Moreover, they are self-constituting (self-producing and self-distinguishing), depend on active engagements with the external enabling relations, and can exist at different scales from metabolic and immune system activity to nervous, sensorimotor, and social dynamics (Arandia and Di Paolo, 2021). The most basic form of autonomy can be seen in *autopoiesis*—the capacity of living systems to generate and keep their identity as distinct from the environment (Segundo-Ortin, 2020). Autopoiesis and adaptivity are critical to the self-organization and autonomy of living beings (Varela, 1997; De Haan, 2020b). According to Di Paolo (2005, p. 439) “*Autopoiesis provides a self-distinct physical system that can be the centre of a perspective on the world, and a self-maintained, precarious network of processes that generates an either-or normative condition. Adaptivity allows the system to appreciate its encounters with respect to this condition, its own death, in a graded and relational manner while it is still alive*.”. From an osteopathic perspective, it can be argued that the concepts of autonomy, autopoiesis and adaptivity are fully aligned with the concepts of body-mind unity, self-regulation and adaptation.

Another fundamental notion of enactivism is *sense-making*—the evaluative interaction between an organism and its environment (De Haan, 2020a,b). More formally, *sense-making* is defined as the capacity of an autonomous system to regulate its operation adaptively and its relationship with the environment depending on the virtual consequences for its viability as a form of life (Di Paolo et al., 2018). Sense-making is, therefore, critical to living, is embodied and embedded, implies values (metabolic), is affective, and can be reflexive (existential sense-making) (De Haan, 2020a,b). The combination of precarious autonomy, sense-making, and adaptivity underpin perception, action, emotion and cognition (Arandia and Di Paolo, 2021). Being a sense-maker implies being ready to selectively act on the affordances provided by the environment, which are relevant to maintain autonomy—sense-making is paramount to understand agency (Segundo-Ortin, 2020). The enactive approach interprets organisms as co-arising and co-determining with their (social) environment. Arguably, the clinical encounter can be seen as a form of social environment, and the development of a robust therapeutic alliance can be regarded as a form of *participatory sense-making*. Participatory sense-making is defined as “*the coordination of intentional activity in interaction, whereby individual sense-making processes are affected and new domains of social sense-making can be generated that were not available to each individual on her own*.” (De Jaegher and Di Paolo, 2007, p. 497). Meaning emerges through these interactions, and it is linked to embodied agency and experience, history, and situation, with sensorimotor agency arising from perception-action loops. Investigating how these loops contribute to the development of a solid therapeutic alliance, how they are affected by pain and disability considering the three entangled dimensions of embodiment (organic/physiological, sensorimotor/psychological and intersubjective/social), and how to develop strategies for increasing part of the agency lost is crucial to person-centred osteopathic care.

From an enactivist standpoint, therapeutic interventions may be used to increase insight, i.e., helping patients recognise what they are feeling and their dominant patterns of sense-making (De Haan, 2020b). The clinical encounter provides opportunities to recognise inflexible and inappropriate patterns of interaction and rehearse different strategies to engage with the world, oneself and others, in what can be described as *participatory sense-making* (De Haan, 2020a).

## Active Inference

Osteopaths seek to understand the nature of their patient’s breakdown in adaptive capacity to inform how osteopathy may improve their adaptability and, therefore, their return to health and wellbeing. Arguably, some of the osteopathic core concepts—unity, self-regulation and adaptation—align with the enactivist account, namely with the concepts of autopoiesis and sense-making. Although enactivists argue that autopoiesis and sense-making are central to understanding basic and non-basic minds, it is also critical to understand the continuity between life and mind through the lens of the free energy principle (FEP) (Wiese and Friston, 2021). The FEP provides a formal analysis of the properties central to autopoiesis and sense-making with active inference—and is instantiated by all systems with a Markov Blanket (Wiese and Friston, 2021).

Under the FEP, dynamical systems, given sufficiently stable boundary conditions, spontaneously self-organize to minimize their expected variational free energy, effectively maximizing evidence for the world model they enact (Friston et al., 2013). The autonomy of living systems is regarded as necessary condition that allows their behaviour described in terms of inference and free energy minimization (Allen and Friston, 2018; Kiverstein, 2020). Although FEP fully endorses the Bayesian brain hypothesis, the only way we can alter the shape of things, i.e., bound entropy production, is to act on the world—this is what distinguishes the FEP from predictive coding (Friston et al., 2018). Therefore, through active inference—i.e., through perception-action loops in which the organism engages with the relevant affordances of its environment—that the organism maintains a distinction between itself and the environment (Kiverstein, 2020). The organism just is an actively maintained boundary—technically known as a Markov Blanket—separating the external environment from its internal dynamics through active inference (Friston et al., 2013; Hohwy, 2016; Kiverstein, 2020). Markov Blankets mediate the exchange between internal and external states and thereby imply perception-action loops, as the organism engages with the relevant affordances of its environment (Kiverstein, 2020). Crucially, active inference is not simply a description of the embodied brain reducing the uncertainty about its sensory observations *via* perceptual inference—it concerns the active and selective sampling of the world by an embodied agent (Ramstead et al., 2019). Creatures are open, complex dynamic systems and changes to one aspect of the self are likely to result in eddies and ripples of disorder, that might lead to a person becoming a patient. Understanding the FEP—as foundational for active inference—is therefore central to our thesis.

### Active Inference and Generative Models - Free Energy Principle and Markov Blankets

According to the FEP, any adaptive change made by a biological system or organism must minimize its long-term average surprise, i.e., its unpredicted sensations or prediction errors (Edwards et al., 2012). Organisms must therefore behave in a way that maintains themselves in the low entropy states they expect to be in. This is achieved by minimizing a measurable approximation to the entropy of these states, i.e., free energy (Seth, 2021). A failure to minimize free energy would lead to a progressive increase in entropy (sensory disorder) and violate the principles of self-organization and homoeostasis (Edwards et al., 2012). As noted above, minimizing free energy or prediction errors requires the organism to have a hierarchical generative model of how sensations (exteroceptive, interoceptive and proprioceptive) are caused (Edwards et al., 2012; Seth, 2021). Biological systems then use these models to reduce the difference between predicted and actual sensory signals, either by changing sensory samples through action—*active inference*—or by changing the predictions through perception—*perceptual inference* (Edwards et al., 2012; Friston et al., 2013; Seth, 2021). Through the lens of FEP, predictive coding, active and interoceptive inference can, in their entirety, be understood as flowing from a critical constraint on what it means to be alive, on what it means to exist (Seth, 2021).

Active inference is a “first principles” approach to understanding sentient behaviour—perception, planning, and action in terms of probabilistic inference—framed as a single imperative to minimize free energy (Parr et al., 2022). The FEP reflects the enactivist account of cognition—embedded, extended, embodied, and enactive. Importantly, active inference is about the consequences of action in the future. Therefore, it goes beyond predictive coding. The objective is not simply to use a generative model to infer the latent or hidden states that cause sensations but to act in a way that minimizes expected surprise, i.e., entropy or uncertainty (Friston et al., 2020). In short, in active inference, the agent adjusts to their environment by acting upon or changing the world to bring about the state of the world predicted by the current best generative model (Venter, 2021).

**FIGURE 2:**
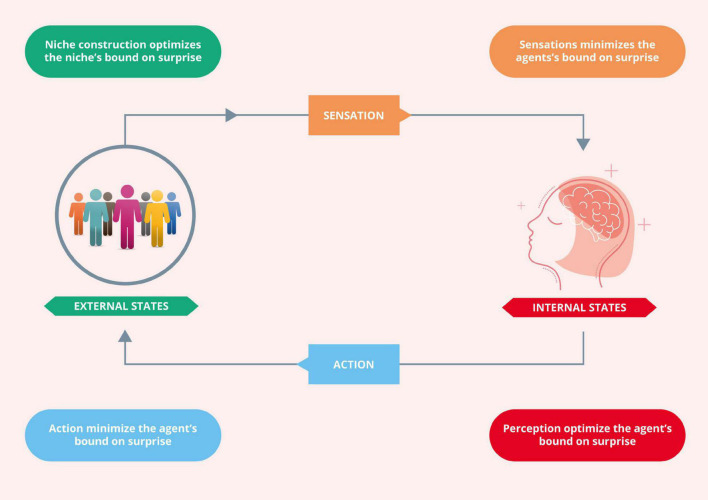
Active inference. This Figure depicts the coupling of an agent’s internal states (the dynamics of which entail predictions or beliefs about the niche) to its external states (the dynamics of the agent’s niche). Adapted with permission from Vasil et al. (2020).

The FEP is a principle of least action, which describes dynamics in terms of the most likely paths any system will take. It entails separating the states of some universe into the (internal) states owned by an agent and those (external) states that are not, and the (blanket) states that mediate the exchange between them. Self-organizing systems are attracted to certain states or specific paths, and this attraction can be described in terms of active inference. In active inference, the self-organizing system corresponds to the *internal states*, which *via* the *active* and *sensory states* affect and are affected by the environment, i.e., the *external states* (Figure 2). Ultimately, a system that self-organizes is a system that minimizes free energy (Parr, 2020; Parr et al., 2020; Hipólito et al., 2021). The relationship between active inference and FEP is operationally simple—active inference is the application of the FEP to a particular system. Importantly, when talking about self-organizing systems, we should not just talk about an organism or agent but also about, for example, the interoceptive or the neuroendocrine-immune systems. These systems, which are crucial to understanding adaptation and osteopathic care, can be conceived as self-organizing systems with their local environment, states, and Markov Blankets.

In summary, to exist, a system needs to have a degree of independence from its embedding environment (Ramstead et al., 2020a,b). A Markov Blanket (or Markov boundary) is a set of states that separate the system’s internal states from its embedding environment and mediate the interactions between the inside and outside, thus inducing conditional independence between internal and external variables. The absence of certain connections defines the Markov Blanket: internal states do not cause sensory states, and external states do not cause active states (Ramstead et al., 2020a,b). Systems that possess a Markov Blanket must have an implicit generative model. The generative model is the only construct needed to describe self-organization, where the systems dynamics or can be read as belief updating. Technically, the free-energy and its generative model are not evaluated or realized explicitly (Friston, 2012; Ramstead et al., 2019, 2020b). This is because the dynamics of the internal states are driven by free energy *gradients*. These free energy gradients are the prediction errors above.

### Active Inference as Enactive Inference- Shared Intentionally, Ecological Niches and Affordances

The organism, its body and brain, and the world constitute the generative model. This can be seen as analogous to an *Umwelt*, in which an organism’s world is a key component of its embodiment (Allen and Friston, 2018). It can therefore be argued that conscious experience is shaped by active inference and rooted in embodied activity, coupling the agent—an embodiment of a generative model—to the world (Kirchhoff and Kiverstein, 2019). This generative model underwrites the organism’s interactions with its environment to ensure the maintenance of a robust brain-body-environment system (Bruineberg et al., 2018). Under active inference, the generative model itself never exists outside the organism’s adaptive actions and policy selections. Therefore, the generative model can be regarded as being enacted (Ramstead et al., 2020b). This view is aligned with embodied and enactive approaches to cognition and enables one to model the dialectic between embodiment (what an organism is) and enactment (what an organism does). On this view, the generative model is what the organism expects and guides what the organism is and does (Ramstead et al., 2020b). Active inference can therefore be viewed as enactive inference (Ramstead et al., 2020b). (En)active inference is the process by which dynamical systems autonomously enact adaptive agency (Ramstead et al., 2020b).

**FIGURE 3:**
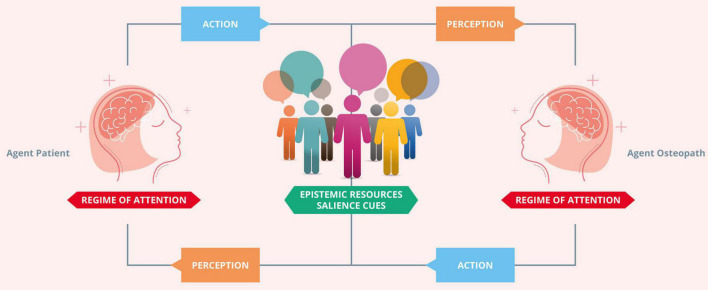
Thinking through other minds. The agent osteopath and the agent patient build shared expectations through engagement with the clinical encounter social and material affordances, allowing adaptive niche construction by thinking through other minds. In the context of communication in clinical practice, coupled dynamics are strengthened by an adaptive prior for alignment. The adaptive prior for alignment specifies the characteristically enhanced precision of the hypothesis that “we” exist. This prior motivates similar agents to actively couple their respective actions and perceptions. The coupling of perception and action enables the osteopath and patient to reliably align with (i.e., infer) the hidden states of the other. This circular process brings about a process of cultural niche construction that creates, maintains, and modifies a set of predictable epistemic resources. These specify a set of high value (i.e., predictable) observation-policy mappings, which are used to disambiguate the mental states of conspecifics. Effective communication plays a crucial role in enabling agents with an adaptive prior for alignment to effectively disambiguate external states. An agent’s external states are constituted, in part, by the internal, mental states of another agent (and vice versa). This follows from the fact that external states cause sensation; for an agent equipped with an adaptive prior for alignment, inferring external states entails inferring other agents’ hidden states. Adapted with permission from Vasil et al. (2020).

Active inference enables an organism or an agent to adjust to their environment to fit their expectations, and therefore construct their niche (Bruineberg et al., 2018; Constant et al., 2018). In particular, agents build shared expectations through engagement with everyday social and material affordances, allowing adaptive niche construction by, for example, thinking through other minds (Laland et al., 2001; Boyd et al., 2011; Veissière et al., 2020; Figure 3). Crucially, cognition is ecological, and as such, it depends on the affordances for action provided by the environment, i.e., the field of affordances (Rietveld et al., 2018). From a clinical perspective, the ecological-enactive and active inference frameworks are nicely exemplified by the treatment of chronic pain above: pain underwrites embodied action that reflects the uncertainty of body and world (Tabor et al., 2017; Miyahara, 2021), which ultimately alters the interaction of the organism or agent with the environment and, therefore, the fields of affordances either temporarily (acute pain) or long term in persistent pain (Coninx and Stilwell, 2021).

## Osteopathic Care as (En)Active Inference

Osteopathic care can be considered in terms of inference about others, based on the notion that we model and predict our sensations—sensations that other agents like ourselves generate. This viewpoint leads to osteopathic care based on a generative model or narrative shared by agents who exchange sensory signals. The dyadic or participatory sense-making process is informed by selectively attending and attenuating sensory information. Attending to interoceptive, exteroceptive and proprioceptive sensations enables agents to predict each other’s sensory input. Conversely, attenuating relevant interoceptive and exteroceptive input enables one to articulate the narrative by realising proprioceptive predictions (e.g., movement). The mental states—hidden states of patients are not observable, and they need to be inferred, and, arguably, osteopaths achieve this through communication, touch, movement and exercise. The dyadic relationship leads to forming an ecological niche beyond the body itself, where alignment is fundamental. In what follows, we build on the concepts introduced earlier to propose an (En)active inference account for osteopathic care. It can be argued that our proposed framework is not unique to osteopathy but can in fact, inform clinical practice in other healthcare professions such as physiotherapy and chiropractic, where touch and manual therapy play a central role in patient evaluation and care. Arguably, some of the differences in patient evaluation and care may be related to the various professions’ theoretical and philosophical conceptual frameworks. Although our proposals have implications for patient care in other professions, we centre our discussions on osteopathic care for the purpose of this paper.

### Osteopathic Care as Participatory Sense-Making, Shared Expectations, Synchronisation and Shared Generative Models

One can regard osteopathic care as an interactive process through which the two complex, dynamic systems (i.e., patient and practitioner)—with the multilevel Markov Blankets—working together to establish shared narratives (including body narratives). Contextual factors and verbal and non-verbal communication strategies within the patient-practitioner dyad—language and hands-on care—are bidirectional sign-vehicles of active (providing sensory data) and sensory states (receiving information). Hands-on strategies in osteopathy and other healthcare professions are a unique form of communication, which can be regarded as an interactive action-perception dynamic loop of the two active inference systems.

From a clinical application standpoint, to understand and support, for example, those suffering with persistent physical symptoms, osteopaths should consciously create new priors or alternative hypotheses for predicting sensory data and establish new ways of sense-making. Arguably, our proposal has implications for other healthcare professionals (e.g., physiotherapists and nurses) who are typically involved in the care of individuals who have musculoskeletal disorders (Rossettini et al., 2018; Palese et al., 2019). Operationally, clinicians should, therefore, recognise inflexible and inappropriate patterns of interaction and implement clinical strategies for their patients to engage with the world—all depending on the context of their symptoms and their personality (Von Mohr and Fotopoulou, 2018). We see osteopathic care, and musculoskeletal care in general, as a dynamic interactive ritual that affords opportunities for reinterpreting sensory signals, redeploying attentions, and attenuating and ignoring the irrelevant sensory inputs. To this end, osteopathic and musculoskeletal care should be conceived as participatory sense-making (De Haan, 2020a).

Effective communication, particularly touch-based strategies, can establish the foundation for trust, compliance, cooperative and prosocial communication (Morrison et al., 2010). Crucially, hands-on care provides an effective vehicle for conveying one’s perceptions and thoughts, providing context and clarity, and establishing interpersonal connection as well as inferring another’s sensory, emotional, and mental states, thus facilitating biobehavioural synchrony (Hertenstein et al., 2006; Mc Parlin et al., 2022). The bidirectional response elicited by hands-on and supported by effective communication provides context for the sensory stimuli by matching with the practitioner’s intention to improve their patient’s symptoms, allostatic regulation, and homeostatic control during treatment (Ackerley et al., 2014; Song et al., 2021; Mc Parlin et al., 2022). We would argue that osteopathic care underpinned by a robust therapeutic alliance may facilitate the development of brain-to-brain synchrony (Morrison et al., 2010), allostatic co-regulation (Atzil-Slonim and Tschacher, 2020; Nguyen T. et al., 2021; Mc Parlin et al., 2022), and therefore consolidating interpersonal relationships (Shamay-Tsoory et al., 2019). Establishing synchrony is crucial to the osteopathic clinical encounter because the multilevel shared Markov Blankets—with mutually predictive sensations generated by the two agents—provide the grounds for a shared narrative and epistemic trust, which are necessary to dissolve strong beliefs and priors. In short, because *you are me and I am you—and therefore I can trust you, I believe my explanations and my narrative about me, and if you are me, then your explanations, your narrative about you must be fit for purpose to explain me.* This also has substantial implications for musculoskeletal care in general—patients regard hands-on care as a fundamental element of the patient-practitioner relationship, thus strongly requesting them (Rossettini et al., 2018). Therefore, establishing synchrony provides multiagent pro-dyad exchange within an established ecological niche, where agents share a sensorium, a common language, a narrative or a generative model.

### ‘Self-Flattening’ and Psychologically Informed Osteopathic Care

As person-centred care, it is crucial that osteopaths shift away from body-centred passive models of care, to consciously consider the role of effective communication, education and reassurance—this could be conceived as psychologically informed osteopathic care. From an (En)active inference perspective, psychologically informed osteopathic care works bidirectionally—in providing the novel sensory data, hands-on care influences the predictive processes from lower to higher levels of hierarchical neuronal architectures, and in revising the implicit generative models (new interpretations and intentions), communication, education and reassurance influences the predictive processes from higher to lower levels. In this context, sensory signals caused by hands-on care strategies will be instantly, and possibly self-evidently, interpreted at the lower levels, with the reference to the generative models being revised by the clinician’s narrative. This can be regarded as “flattening sensory data,” i.e., by adapting Limanowski and Friston’s (2020) concept of “self-flattening”. This is, arguably, the process by which psychologically informed osteopathic care modifies the active inference cycle by providing a new way of sense-making with the patient’s selfhood by selectively attending to interoceptive, exteroceptive and proprioceptive sensations. It is therefore important to both attenuate some forms of attention that are orchestrated by the patient’s higher levels of their hierarchical generative models. We would argue that robust psychologically informed osteopathic care may provide patients with something like deep mindful meditation experiences, which flatten the landscape of free energy through reducing the precision of high-level prediction errors—and implicitly the top-down deployment of precision or attention to sensory prediction errors.

In a state of health and wellbeing, our bodies are typically invisible. In the case of pain and dysfunction our attention is drawn toward our bodies, something that can be explained as a failure of sensory attenuation. What was invisible and now rendered visible, mandates for some explanation that we are compelled to make sense of our world and thus finding an explanation for this sensory evidence. Arguably, one has lost the ability to switch on and off, that otherwise would have rendered things invisible. Paradoxically, this is not a failure of attention, but a failure of *dis-attention*. It is a failure in one’s ability to ignore, and one is therefore compelled to find explanations for their personal experience. For example, when applied to interoception, one can link this inability to attenuate precision of prediction errors to hypersensitivity in terms of interoceptive accuracy.

From an osteopathic standpoint, we need to equip the patient, or restore the patient’s ability to ignore, or at least to have some volitional control to what they attend to. Directing the patient’s attention to different parts of their sensorium, and directing it away from other aspects of their belief system or their hierarchical generative model will enable the patient to revise their model through perceptual and active inference. Ultimately, osteopathic care may update interoceptive active inference processes by updating the sensory inputs providing action-executions (allostatic prediction errors) and therefore bringing the system to update the homeostatic forecasting model (Bohlen et al., 2021; Petzschner et al., 2021). This means that patients have to learn new ways of attending to—and attenuating—proprioceptive and interoceptive signals, which speaks to the fundamental importance of ‘hands-on’ therapies that supply such signals. For further insights into the practical application of this theoretical framework, the reader is directed to our research hypothesis and theory article on osteopathy and mental health (Bohlen et al., 2021).

## Future Directions—Osteopathic Care and Beyond

In this paper, we have proposed a theoretical framework for developing an integrative hypothesis in osteopathy, which is based on the enactivist and active inference accounts. This theoretical framework has implications for osteopathic care across the lifespan and with different patient populations. In situations of chronicity, osteopathic care—underpinned by a robust therapeutic alliance—enables patients to make sense of their illness experiences by creating new narratives (including body narratives) about their changed or changing physical capacities, with ensuing effects on their identity, relationship with their environment and meaning in their lives. From an (En)active inference perspective, the dyadic participatory sense-making within a shared ecological niche underwrites a robust therapeutic alliance, which is crucial to the effectiveness of osteopathic care. In the fields of neonatology and paediatrics, touch becomes central in developing a robust therapeutic alliance, mental state alignment, and biobehavioural synchrony between patient and practitioner —all crucial in regulating allostasis, restoring homeostasis and addressing the clinical problem.

This framework has implications to osteopathic care, education and research, which may apply to other models of care in the broader field of musculoskeletal and psychologically informed care (e.g., physiotherapy, chiropractic and nursing). To this end, we prioritise several areas for future research including the investigation of the mechanisms underpinning the process of making sense of bodily signals and the effects of osteopathic care, and manual therapy in general, on those mechanisms in two clinical populations: individuals suffering from chronic pain and other persistent physical symptoms, and preterm infants. This line of research focuses on the precision, sensory attenuation, and mental action for deploying attention at different levels in a very hierarchical generative model as a means of gating the sensory evidence that one has to explain.

Chronic pain and persistent bodily symptoms are associated with an inability to ignore, attend away or selectively attenuate different sources of sensory evidence. Little is known regarding the effects of osteopathic care on these mechanisms. We propose research investigating the role of osteopathic care in enabling patients with persistent pain and bodily symptoms to deploy precision, augment or attenuate it in the context of selective attention and selective sensory attenuation. This line of research should investigate the role of osteopathic care and manual therapy in enabling individuals with these chronic conditions to regain their sense of agency. Research methods may include neurocomputational models, neuroimaging studies, clinical trials, experimental studies and qualitative phenomenological studies.

Prematurity is linked to failures in the development of the minimal and social self and in the ability to selectively attend or attenuate to affective touch and other forms of nurturing behaviour. Our current research demonstrates that osteopathic care (Lanaro et al., 2017; Manzotti et al., 2020) and affective touch (Manzotti et al., 2019) play a positive role in the health and wellbeing of preterm infants in a neonatal intensive care setting. However, the effects of osteopathic care, manual therapy, and affective touch on the neurodevelopment of premature infants are currently unknown. We propose research investigating how early osteopathic care, manual therapy and affective touch in neonatal intensive care settings may contribute to the neurodevelopment of prematurely born infants. This research should focus, particularly, on the continuum between the development of the minimal self and the interactive, social self. Research methods may include clinical trials, experimental studies, cohort studies and neurocomputational models.

In addition to researching the mechanisms and effects of osteopathic care, affective touch, and manual therapy, we propose exploring the interdependent processes within and between practitioner and patient in a dynamic dyadic therapeutic interaction. To this end, we propose research investigating the interdependent processes within and between practitioner and patient in a dynamic dyadic therapeutic interaction in osteopathy, paediatrics, and musculoskeletal care. To fully understand the mechanisms underpinning osteopathic care and manual therapy in different clinical populations, it is crucial to study treatments as dynamic systems, focusing on processes within the patient, within the practitioner and between patient and practitioner (i.e., the dyad). This line of research should focus primarily on dynamic dyadic processes such as synchrony and co-regulation. To this end, we propose studying objective physiological and subjective (experienced) processes, including their interrelationship across different time scales. This line of research will ultimately contribute to understanding the underlying mechanisms of existing therapeutic interventions in osteopathy and manual therapy (and the development of new interventions), which can improve the patients’ health and well-being through dynamic dyadic processes. The premise of these investigations is that both practitioner and patient are trying to render themselves mutually predictable and that therapeutic efficacy (e.g., based on establishing epistemic trust) will be reflected in peripheral markers of mutual predictability afforded by measures of generalised synchrony (e.g., in video kinetics, ECG, heart rate variability, pupillometry, EEG, respiration).

## Conclusion

Here, we have proposed a theoretical account for developing an integrative hypothesis in osteopathy under the (En)active inference framework. This paper does not propose a new osteopathic model of care. Instead, the theoretical framework presented here lays the foundations for developing and validating an integrative model for osteopathic care, which can inform professional practice in a diverse range of clinical populations. In particular, the proposed framework can inform clinical practice and research in the broader concept of psychologically informed osteopathic care. (En)Active inference offers an integrative framework for osteopathy, which can evince the mechanisms underpinning dyadic exchanges and osteopathic care outcomes. As an ecological niche, the patient-practitioner dyad provides the osteopath and the patient with a set of affordances that can promote adaptations and restoration of productive selfhood. For example, in the context of persistent physical symptoms associated with a range of emotional and cognitive factors, osteopaths need to enable patients to increase their repertoire of attentional deployment to a level beyond the re-interpretation of their interoceptive signals. Instead, they should enable patients to restore their ability to ignore and reappraise irrelevant signals, as means of returning to a natural state of rendering, where appropriate, things invisible. This should equip patients with the ability to regain control and explore other ways to attend to relevant signals from within their body. Crucially, to enable patients to meaningfully interpret these sensory signals and therefore revise their generative models, clinicians should use non-threatening, non-nocebic language. We argue that the clinical encounter provides opportunities to identify maladaptive priors and beliefs and implement strategies to engage with the world as participatory sense-making. The theoretical framework presented in this paper provides osteopaths and other healthcare professionals with a “first-principles” approach to understanding their patient’s unique “story” and how different bio-psycho-social factors interact to contribute to their bodily experiences and narratives. To provide truly person-centred care, practitioners must move away from dualistic thinking to understand how patients’ interactions with their environment affect their experiences, expectations, and beliefs. In the case of persistent pain and other physical symptoms, how they contribute to their suffering. We argue that manual therapy is still an essential aspect of osteopathic and musculoskeletal care; however, it should be used wisely within a person-centred model where contextual factors, language, reassurance, education, and non-verbal communication are effectively considered and used.

## Author Contributions

JE wrote the first draft of the manuscript. All authors contributed to conception and revision of the manuscript, and approved and are accountable for the submitted manuscript.

## Conflict of Interest

FC and JE are Topic Editors for the Research Topic: “Enactivism and Active Inference in the Therapeutic Alliance” but were not involved in the review or approval of this manuscript. The remaining authors declare that the research was conducted in the absence of any commercial or financial relationships that could be construed as a potential conflict of interest. The handling editor declared a past co-authorship with JE and FC.

## Publisher’s Note

All claims expressed in this article are solely those of the authors and do not necessarily represent those of their affiliated organizations, or those of the publisher, the editors and the reviewers. Any product that may be evaluated in this article, or claim that may be made by its manufacturer, is not guaranteed or endorsed by the publisher.

## Footnotes

1 Strictly speaking, the free energy principle says that systems that resist a tendency to disorder must counter dissipative fluctuations by, on average, decreasing surprisal.
